# Adolescent girls' perception about their ability to safely offer HIV self-test kits to sexual partners: a feasibility study in Siaya County, Kenya

**DOI:** 10.4314/ahs.v21i3.13

**Published:** 2021-09

**Authors:** Gift-Noelle Wango, Kawango Agot, Henry Ogolla, Marylyn Ochillo, Spala Ohaga, Harsha Thirumurthy

**Affiliations:** 1 Nyanza Initiative for Girls Education and Empowerment, Kisumu, Kenya; 2 Impact Research and Development Organization, Kisumu, Kenya; 3 Department of Medical Ethics and Health Policy, Perelman School of Medicine, University of Pennsylvania, Philadelphia, USA

**Keywords:** HIV self-testing, partner testing, couples testing, adolescent girls, Kenya

## Abstract

**Background:**

Adolescent girls (AG) in sub-Saharan Africa are at elevated risk of acquiring HIV, yet few know the HIV status of their sexual partners. Interventions to promote testing among partners are urgently needed.

**Objectives:**

To explore AG's perceived ability to safely distribute HIV self-tests to their partners, if partners would self-test, and how to minimize partner violence.

**Methods:**

We recruited HIV-negative AG ages 15–19 years with a partner of unknown HIV status or who tested negative >6 months previously. Using mixed-methods for data collection and regression and inductive thematic analysis for quantitative and qualitative analysis, respectively, we determined factors associated with the study objectives.

**Results:**

We enrolled 101 AG, median age 17.3 years, sexual debut 15–16 years, and 54.5% reported ≥2 lifetime partners. Most participants (95.0%) would offer self-tests to their partners and 95.1% reported high-to-moderate chance their partner would self-test. No participant attribute was associated with perceived ability to offer self-test or likelihood of partner testing. To avoid violence, AG recommended politeness, indirect approach, voluntariness, and highlighting advantages of self-testing.

**Conclusions:**

AG believe they can safely distribute self-tests to their partners, and most partners would self-test, expanding utility of HIV self-tests to include partners of AG.

## Introduction

Adolescent girls and young women (AGYW) make up only 11% of the adult population yet account for 20% of new HIV infections globally^1^. Despite a large decline in new HIV infections in eastern and southern Africa from 2005–2015, progress has slowed in recent years and most alarmingly, HIV risk among AGYW in some regions remain extremely high.^2^–^4^ Transactional sex -often in age-disparate relationships – is believed to be among the main driving factors for the HIV risk in this population^2^. One of the five prevention pillars identified by UNAIDS for progress towards the 90-90-90 targets by 2020 includes access to services for not only adolescent girls but also their male partners^1^. However, achieving the first 90 is undermined by the fact that only 23% of adolescent girls know the HIV status of their partners, with those who do not being 14 times more likely to be infected than those who know^5^.

A promising strategy for closing the testing gap among adolescent girls (AG) and their male partners is HIV self-testing (HIVST), an approach to HIV testing that has gained prominence in recent years. A number of studies have documented that HIVST has high acceptability in diverse populations and settings^4^,^6^,^7^,^8^ Several recent studies have drawn attention to the potential for HIVST to facilitate male partner testing through secondary distribution of self-test (where index clients are given multiple self-tests to distribute to those in their sexual networks) from women to men.^14^–^16^ To our knowledge, no studies have directly explored whether secondary distribution of self-tests by AG to their partners is an acceptable, feasible and safe approach to increase awareness of partner status, and possibly identify HIV-positive men. However, the approach may also carry risks of intimate partner violence (IPV) especially in an age-disparate relationship as AG may lack the agency to initiate HIV testing discussions with their partners. It is therefore important to gauge AG's perceived ability to offer self-tests to their partners and whether they believe the partners would self-test, before they are given actual test-kits to distribute to their partners.

## Methods

### Study setting, design and participants

the study was done in siaya county where Impact Research and Development Organization's (IRDO's) HIV testing activities were being implemented. Siaya County currently has the third highest HIV prevalence in Kenya, estimated to be 15.3% in the general population^17^. We conducted a feasibility study to plan for a larger randomized trial in which adolescent girls age 15–19 years would be given multiple self-tests for own use and distribution to male partners, or multiple referral cards designed to encourage male partners to seek provider-assisted HIV testing services. The purpose of the feasibility study was to determine if AG perceived themselves as capable of offering self-tests or conventional HTS referral cards to their partners, and to collect their views on strategies to avoid conflict with partners. The study was approved by Maseno University Ethics Review Committee (Ref: MSU/DRPI/00247/15).

During the study, IRDO's HTS Counsellors providing testing in communities in Siaya County referred all AG aged 15–19 years (lowest age of individual consent for HIV testing in Kenya is 15 years^18^) who tested for HIV. Eligibility for the study included: presenting a referral card from IRDO confirming testing for HIV in the previous three months, age 15–19 years, living in Siaya County, reporting ≥1 sexual partner with whom they had sex in the past 6 months, intending to meet the partner at least twice in the next 3 months (to allow time for offering the test-kit or referral card), having a partner of unknown HIV status or tested HIV negative >6 months ago, not worried that the partner may harm them if offered self-test kit or referral card, and willing to give written informed consent.

### Data collection

Eligible, consented participants received basic information about HIVST and a demonstration on how the kit is used; they were however not issued with kits to take home. The participants were then taken through a paper-based, interviewer-administered structured questionnaire on their health and sexual behaviors, their partner's HIV testing history, their perception of their ability to offer self-tests or referral cards to their sexual partners, and their perception about the likelihood of their partners testing or harming them. The questionnaire also contained open-ended qualitative questions to explore reasons for selected responses, with one asking: “How can you advise us to make it safe for girls who enroll in this study to not be harmed by their partners if they offer them self-test kits and encourage them to test?,” which allowed us to document participants' recommendations on how to make it safe for AG to offer self-tests to their partners. Other open-ended qualitative questions explored how participants would address challenges in offering HIVST kits to their partners and what would make it easy or difficult to introduce HIVST to partners.

### Measuring quantitative responses

Most responses to socio-demographic, health and sexual behavior, and history of HIV testing questions were measured in actual numbers reported (e.g., lifetime sexual partners or number of times tested for HIV in the past 12 months), as yes or no (e.g., if have children or if partner would be angry if offered an HIV test at home), or as selection from multiple-choice options (e.g., marital status or level of schooling attained). Questions that sought participants' perception on ability to offer HIVST were measured using a 5-point Likert scale (very easy, somewhat easy, neither easy nor difficult, somewhat difficult, very difficult) or simple ranking (high chance, moderate chance, low chance, no chance).

### Study outcomes

The primary outcome was the proportion of AG reporting being able to offer self-test or referral card to their partner, and secondary outcomes were: i) the proportion of AG reporting that their partner would use the self-test kit or referral card to test, and ii) recommendations on how AG can minimize the likelihood of IPV while distributing test-kits for partner and couple testing.

### Data analysis

We generated descriptive statistics of participants' sociodemographic characteristics, health and sexual behavior, partner's HIV testing history, and AG's perceived ability to offer self-tests or referral cards to their male sexual partners. Cross sectional regression or multiple variables regression was performed to control for potential confounding factors and determine the contribution of dependent variables to outcomes (chance partners would test and chance for couple testing). A p-value of <0.1 was considered significant in MR models. All analyses were performed using Stata 14.0 (StataCorp). For qualitative analyses, participants' responses were transcribed and where relevant, translated from the local language (Kiswahili or Dholuo) into English. Common responses were identified and tallied by the number of participants who mentioned them. Using an inductive thematic analysis approach^20^, data were analyzed to identify core theoretical concepts, themes and patterns, with most giving more than one recommendation.

## Results

### Participant characteristics

Between August 2016 and February 2017, we enrolled 101 AG from 148 referrals. Forty-seven were excluded for: having no sexual partner in the previous six months (n=24), not planning to meet the partner at least twice within three months (n=9), intending to relocate outside Siaya County (n=5), younger than 15 years or older than 19 years (n=3), partner tested negative within six months (n=3), concerned about possible violence from partner (n=2), or forgot to carry the referral card from IRDO (n=1).

The median age of participants was 17.3 years (IQR=16–18 years), 82.2% were unmarried, and 61.4% had no children (Table 1). A majority (60.4%) reported having completed secondary school and over half (54.5%) reported having ≥2 lifetime partners, with 24.8% reporting ≥3 lifetime partners. Majority (47.5%) started having sex at age 15 or 16 years, with about 9% initiating sex by or before age 12. Half (50.5%) reported having the first sexual encounter with their age-mates, 42.6% by those older by <5 years and only 5% reported that their partner was ≥5 years older.

**Table 1:** Socio-demographic and selected sexual behavioral characteristics of participants

Parameter	Frequency (%)
**Age in years, median (IQR)**	17.3 (16–18)
**Age, N (%)**	
15	10 (9.8)
16	21 (20.8)
17	22 (21.8)
18	24 (23.8)
19	24 (23.8)
**Education level, N (%)**	
Any primary school	40 (39.6)
Any secondary school	59 (58.4)
Post-secondary	2 (2.0)
**Marital status, N (%)**	
Not married	83 (82.2)
Married, not living together	184 (4)
Married, living together	14 (13.9)
**Number of children, N (%)**	
None	62 (61.4)
≥ 1	39 (38.6)
**Age at first sex, N (%)**	
Median (IQR)	15 (14–16)
<12	9 (8.9)
13 – 14	22 (21.8)
15 – 16	48 (47.5)
≥17	16 (15.8)
Don't know/Refused to answer	6 (5.9)
**Number of lifetime partners, N (%)**	
1	45 (44.6)
2	30 (29.7)
3	16 (15.8)
≥4	9 (8.9)
Refused to answer	1 (1.0)
**Age of sexual partner, N (%)**	
Younger or same age	51 (50.5)
<5 years older	43 (42.6)
≥5 years older	5 (5.0)
Don't know	2 (2.0)

In cross-sectional analysis, chances that a participant would distribute self-test or referral card to a sexual partner or that the partner or couple would test were not statistically significant with selected participants attributes such as age of participant, age of partner, number of children, level of schooling, marital status, lifetime sexual partners, uptake of HTS by partner, and fear of discussing condom and/or family planning use with main partner.

### Perceived ability to offer self-tests to sexual partners

While all participants had been tested for HIV in the past 3 months (an eligibility criterion), only 60.4% reported that their current partner had ever had an HIV test in their lifetime, 11.9% reported that their partners had never tested, and 27.7% did not know if their partner had ever tested (Table 2). Almost 60% of those that had knowledge of partner lifetime HIV testing reported partner had HIV testing within the last 12 months. Only one participant reported having heard of HIVST prior to the study; however, 95.0% reported moderate to high chance they would offer HIVST kit to their sexual partners (16 of 18 who reported having husbands and 80 of 83 who reported having boyfriends) if trained on how to conduct the test. A very small proportion reported a low chance (2%) or no chance (1%) that their partners would use self-tests. The majority reported high (72.3%) and moderate (22.8%) chance that their partners would self-test if offered the test kit. When disaggregated by age of partner, perceived ability to offer HIVST kits were comparable across different age bands: 96.0% of those reporting same age or younger partners, 95.3% of those reporting partners who were older by <5 years, and 100% of those reporting partners who were older by at least 5 years (the last category had only five participants).

**Table 2:** Sexual partners' HIV testing patterns and views on ability to offer self-tests to partner

	Frequency (%)
**Knowledge of main sexual partner ever gone for an HIV test**	
Yes	61 (60.4)
No	12 (11.9)
Don't know	28 (27.7)
**Knowledge of main sexual partner going for HIV test within last 12** **months (N=61)**	
Yes	38 (59.4)
No	23 (41.6)
**Person to offer HIVST kit (multiple responses allowed)**	
Husband	17 (16.8)
Boyfriend	81 (80.2)
Casual sexual partner	2 (2.0)
Friend	24 (23.8)
Relatives (sister, brother or mother)	47 (46.5)
**Chance participant would give partner self-test**	
High	68 (67.3)
Moderate	28 (27.7)
Low	2 (2.0)
No chance	1 (1.0)
Don't know	1 (1.0)
Refused to answer	1 (1.0)
**How might sexual partner react if he is asked to test with self-test?**	
No concerns	71 (70.3)
Suspicious/Refuse to use	14 (13.0)
Can become violent	2 (2.0)
Don't know	9 (8.9)
Other concerns	5 (5.0)
**Chance sexual partner testing if given self-test kit**	
High	73 (72.3)
Moderate	23 (22.8)
Low	3 (3.0)
No chance	1 (1.0)
Don't know	1 (1.0)

On the question of how they believe their partners would react upon being offered a self-test, 70.3% reported having no concern about the possibility to IPV while 13.0% were concerned that their partners could be suspicious or decline to use the test-kit. Only 2% reported being concerned about IPV.

From the semi-structured interviews, all but four participants mentioned ‘husband’ or ‘boyfriend’ as the person to whom they would offer self-tests. One of the four had never discussed HIV testing previously, two were afraid the partners could harm them, and one was staying away from boyfriend. However, all shared strategies to safely offer sexual partners self-test.

### Strategies for safe introduction of self-tests to their partners

Except for three participants who offered no advice, 97% gave five key recommendations on how to make it safe for girls to offer self-tests to their sexual partners: being polite, friendly and observant of partner's mood (n=60); educating girls on how to approach partner without causing tension (n=28); offering testing without coercion (n=27); discussing with the partner issues around HIV/AIDS and the importance of testing (n=22); and introducing self-tests indirectly (n=20). Other recommendations provided by 5–10% of respondents included: presenting HIVST as more convenient than testing at a health facility or being tested by a provider, impressing on the partner that bringing an HIVST home shows they care about the relationship, trusting the girls' instinct on how their partners would react because they know their partners best, offering to test together with the partner to ease tension or reduce suspicion, and confirming if the girls even want to know the status of their partners. (Names of participants whose direct quotes are included have been changed to protect their identity.)

### Being polite, friendly and observant

The majority of participants (59%) recommended that when offering HIVST to partners, AG need to be gentle and friendly while observing the mood of the partner: “*They can have a gentle discussion on testing with their partners and if he looks harsh they should wait for the right time to introduce the kits*” (Amy, 19, single). Anther respondent, Brenda, 16 years and single, elaborated that: “*Let the adolescent girls monitor the mood of their partners.....When his mood is changing or he starts talking loudly or getting angry, stop and change the topic or leave him alone*.”

### Educate girls on correct approach

About 28% of respondents felt that participants should be educated on strategies of approaching their partners in order to avoid harm. A 17-year-old (Cate, single) advised that: “*By teaching the adolescent girls how to talk to their partners about the importance of knowing their status, the benefit of testing....they are likely to see the need to test*.” Other participants felt that AG could avoid violence if they are endearing: “....*call him sweet names, then inform him about HIVST and even mention that ‘I am afraid of going to test at the hospital, but I have these self-tests, can we test together?*’ (Debbie, 15, single).

### Make testing optional

Over one-quarter of participants (27%) were clear that girls should not force their partners to test; “*Do not force him if he doesn't want, but you can challenge him by telling him that you would prefer to date someone whose [HIV] status you know*” (Essie, age 16, single). One advised: “*I can give him the consent form so that he reads and understands, If he accepts I give him but if he refuses, I stop*” (Flora, 16, single).

### Discuss HIV and importance of testing

Over one-fifth of participants (22%) advised on discussing HIV and the importance of testing first before introducing HIVST: “*They need to wait when the man is relaxed then....sit him down, discuss HIV and ask him if you can give him self-test kits*” (Gloria, 18, single). Another participant (Harriet, 15, single), suggested leading by example: “*They (AG) can test themselves first then give their partners*”.

### Introduce HIVST indirectly

One-fifth recommended that AG should introduce HIVST indirectly: “*They can make a story about how life would be if they knew their status. Then talk about the test kit, how to use it, and ask him if he would want to use it to test and know his status*” (Irene, 17, single). Others felt that giving the partner the contact information of the research staff would make the partner less suspicious: “*Give the girls your contact in case the boyfriends are in doubt they can call and confirm*” (Jen, 16, single).

## Discussion

This study adds to the growing body of research around self-test distribution strategies that are optimal for increasing testing access among hard-to-identify individuals such male partners of AG. The evidence from structured interviews suggest very low awareness of HIVST among AG in the study setting, concurrent with findings from previous studies reporting lack of awareness of HIV self-testing in Kenya^21^. However, there was general consensus on the willingness to accept multiple self-tests and offer them to their sexual partners.

Consistent with prior studies confirming older women were able to test together with their partners^14^–^16^, a substantial majority of the participants in our study believed they would use the kit to test together with their partner. These studies reported that participants who tested as a couple and mutually disclosed their HIV status were more likely than those testing alone to adopt a range of HIV prevention and care behaviors. In addition, interventions involving provision of multiple self-tests have been reported to influence sexual behavior by facilitating more informed, safer sexual decision making among men who have sex with men (MSM)^24^,^25^ and FSW^15^,^16^. From a policy standpoint, the provision of self-test kits to AG for their own use and for secondary distribution to their partners has a substantial appeal not only because it will promote partner testing but also because it helps AG learn their partner's HIV status, besides giving rise to discussions about sexual risk reduction strategies.

The most common concern of secondary distribution of multiple self-tests by women to their partners has been the possibility of IPV^15^,^16^,^26^. When asked if they were willing to suggest HIVST to their partner, only 2 participants foresaw the possibility of IPV occurring. Thus, in support of findings from studies among older women, these results highlight low-level potential for violence and other potential harm from partners to AG resulting from self-test distribution^14^–^16^,^26^,^27^.

However, potential risks associated with HIVST and IPV cannot be ignored during distribution, thus we explored strategies AG can use to avoid or overcome conflict and physical harm from partners. The semi-structured interviews elicited information that can be used in developing counseling messages that would make it safe for AG to offer HIVST to their sexual partners and encourage them to test individually or as a couple.

Findings from this study should be considered in light of limitations. First, this was a pilot study with a small sample-size hence the generalizability of the findings may not extend beyond the study region. Second, we enrolled AG who recently tested for HIV and knew their status, thus the results may not be applicable to those who do not know their HIV status or are more averse to testing – women for whom testing with a partner may also be more challenging. Third, we simply elicited adolescent girls' views of their perception about how their partners would react without offering them self-test kits, thus it is difficult to verify if the study findings will be borne out in practice. And fourth, since the questionnaire was interviewer-administered, there was potential for social desirability bias. However, the findings provide a general sense that AG are willing, and perceive themselves as able, to accept multiple selftests and offer to their sexual partners. Additionally, we believe social-desirability bias was likely not a significant issue because participants were open when answering some very sensitive questions, such as one-third admitting initiating sex before age 15 years, over half admitting having had multiple sexual partners, and two-thirds of unmarried participants reporting having at least one child. To our knowledge, the study represents the first assessment of adolescent girls' perceived ability to undertake secondary distribution of self-kits to their sexual partners. However, such interventions must address concerns over the effect of this testing strategy on stability of relationships.

## Declarations

### Availability of data and material

All data generated and analyzed during this study are included in this published article (and its supplementary information files).
